# Editorial: Viral co-infections: challenges and therapeutic approaches

**DOI:** 10.3389/fmicb.2026.1944312

**Published:** 2026-08-31

**Authors:** Sara Louise Cosby, Anna Rosa Garbuglia

**Affiliations:** 1 Wellcome Wolfson Institute for Experimental Medicine, School of Medicine, Dentistry and Biomedical Sciences, Queen's University Belfast, Belfast, United Kingdom; 2 Virology, Veterinary Sciences Division, Agri-Food and Biosciences Institute, Belfast, United Kingdom; 3 National Institute for Infectious Diseases, Rome, Italy

**Keywords:** bacterial, co-infection, genomics, immunity, influenza, RSV, SARS-CoV-2, therapeutics

## Highlights

- Co-infections are generally uncommon, but they can significantly worsen disease severity, particularly in viral–bacterial combinations or high-risk populations.
- Pathogen interactions and infection order play a critical role in clinical outcomes and immune responses.
- Ongoing surveillance, vaccination strategies, and the development of targeted therapies are essential to address the evolving complexity of co-infections.
- Overall, this Research Topic underscores the need for continued monitoring, integrated prevention strategies, and improved therapeutic approaches to manage complex co-infections.

The articles in this Research Topic shed light on the growing importance of co-infections involving various pathogens, particularly in the context of respiratory viruses, HIV, and emerging infectious diseases. Co-infection with viruses such as SARS-CoV-2, influenza, and respiratory syncytial virus (RSV) can influence disease severity and complicate clinical outcomes, especially when combined with bacterial pathogens or underlying conditions.

A comprehensive analysis of the literature is essential to understanding the global research landscape and guiding future studies. Zhang et al. analyzed major publication databases from 2005 to 2025 to evaluate the status, key hotspots, and clinical advancements in influenza co-infection research. Their findings highlighted the significance of both pediatric populations and viral co-infections. Other studies included in this Research Topic further support the relevance of an in-depth understanding of these critical areas of influenza co-strain, co-viral, or co-bacterial infections. Grubber et al. outlined concerns regarding the possible pressure on hospital systems in the likely event of simultaneous increases in SARS-CoV-2, influenza, and RSV infections, referred to as a “triple-demic.” The authors described how some hospital systems in the United States expanded their inpatient screening policies to test patients for all three viruses simultaneously, regardless of the presence of symptoms. This study gives some important reassurance that co-infection with influenza or RSV is rare and that incidences of hypoxemia and death are similar to those of SARS-CoV-2 alone. Pediatric populations in China were examined by Sun et al. for the circulation of influenza viruses following the COVID-19 pandemic. Their findings identified the predominant influenza strains and highlighted the co-circulation patterns, genetic diversity, and antiviral susceptibility profile of the circulating viruses.

While influenza virus co-infections may be infrequent, combined bacterial or mycoplasma infections are much more common and may give rise to increased symptoms and outcomes. Zhou et al. carried out a retrospective cohort study utilizing data from the Fourth Affiliated Hospital of Soochow University. Panton-Valentine Leukocidin (PVL)-positive, methicillin-susceptible ST22 strains were found to cause fatal outcomes in patients co-infected with influenza A or SARS-CoV-2 and associated with severe leukopenia. Similarly, co-infection with *Mycoplasma pneumoniae* (Mp) is frequently documented. To examine this important but understudied co-infection, Ma et al. established a hamster model with H1N1 (swine flu), which demonstrated that H1N1-infected animals that received a follow-up Mp challenge exhibited significantly more severe histopathological lesions, higher pathogen loads, and dysregulated cytokine responses compared to other infection groups. A comprehensive review by Lei et al. discussed primary mechanisms of Influenza-mediated susceptibility to bacterial infection, with a particular focus on epithelial barrier damage, immune cell dysfunction, and the roles of specific immune cells and their effector pathways in inducing hyperinflammatory responses, along with treatment options. The authors emphasized that an in-depth understanding of the interactions between pathogens and hosts will assist in the development of therapeutics for the prevention and treatment of post-influenza bacterial infections.

Co-infections are a well-established, fundamental area of research for Human Immunodeficiency virus (HIV). The importance of both genomic and immunological approaches to understanding HIV strain recombination, co-infections and vaccine efficacy in HIV-infected individuals is emphasized in two articles in this Research Topic. A study by Ye et al. demonstrated that circulating recombinant forms (CRFs) play a critical role in the global transmission of HIV-1, with further second-generation recombinant viruses, derived from the original CRFs, continually emerging. Furthermore, these CRFs are associated with critical changes in co-receptor tropism. This study strongly supports the importance of sustained genomic surveillance for prevention strategies. Xie et al. examined whether there exists cross-protection from the humoral immune response of immunocompromised populations of people living with HIV to both infection with existing COVID-19 sub-lineages and subsequent COVID vaccines. Their study highlighted the critical need for regular monitoring of immune responses to new COVID vaccines targeting emerging variants in this category of individuals.

In addition to studies of co-infections in human populations, research on co-infection in animal species is an important area of veterinary medicine for understanding and utilizing natural virus models and for surveillance and protection against zoonotic diseases. Bats are natural reservoirs for many viruses, but information on hepatitis viruses is limited. Kui et al. examined the diversity and evolution of bat hepatitis B viruses (BtHBVs) and their co-infection with bat hepatitis D virus (BtHDV) in bats in China and showed that they are natural hosts for both viruses.

Advances in genomic surveillance, immunological studies, and experimental models reveal how pathogen interactions, immune responses, and infection timing shape disease progression. In addition, there remains an urgent need for specific antiviral therapeutics, as exemplified by three papers in this Research Topic. Traditional Chinese Medicine (TCM) is of interest to identify and target new substances not currently used in conventional pharmaceutical treatments. A systematic review by Zeng et al. examined herbal agents documented in classical TCM texts for the treatment of Tianxing fever (closely related to the dengue virus) to guide clinical practice and modern drug development for dengue prevention and treatment. Another review by Liu et al. comprehensively summarized the current status of Respiratory Syncytial Virus monoclonal antibodies and small-molecule antivirals. A third experimental study by Liang et al. examined an alternative therapeutic approach to vaccines for porcine reproductive and respiratory syndrome virus (PRRSV). Mogroside V, a triterpenoid compound (derived from *Siraitia grosvenorii*) that has previously been shown to exhibit diverse biological activities, including antioxidant, anti-inflammatory, and anti-cancer properties, with the capacity to scavenge free radicals and mitigate oxidative stress, was assessed *in vitro*. This compound not only directly inhibited PRRSV replication but also significantly upregulated the gene expression of immunomodulatory cytokines.

